# bootComb—an R package to derive confidence intervals for combinations of independent parameter estimates

**DOI:** 10.1093/ije/dyab049

**Published:** 2021-05-19

**Authors:** Marc YR Henrion

**Affiliations:** 1 Malawi—Liverpool—Wellcome Trust Clinical Research Programme, Queen Elizabeth Central Hospital, Blantyre, Malawi; 2 Department of Clinical Sciences, Liverpool School of Tropical Medicine, Liverpool, UK

**Keywords:** Biostatistics, R, confidence intervals, bootstrap, estimation

## Abstract

**Motivation:**

To address the limits of facility- or study-based estimates, multiple independent parameter estimates may need to be combined. Specific examples include (i) adjusting an incidence rate for healthcare utilisation, (ii) deriving a disease prevalence from a conditional prevalence and the prevalence of the underlying condition, (iii) adjusting a seroprevalence for test sensitivity and specificity. Calculating combined parameter estimates is generally straightforward, but deriving corresponding confidence intervals often is not. bootComb is an R package using parametric bootstrap sampling to derive such intervals.

**Implementation:**

bootComb is a package for the statistical computation environment R.

**General features:**

Apart from a function returning confidence intervals for parameters combined from several independent estimates, bootComb provides auxiliary functions for 6 common distributions (beta, normal, exponential, gamma, Poisson and negative binomial) to derive best-fit distributions for parameters given their reported confidence intervals.

**Availability:**

bootComb is available from the Comprehensive R Archive Network (https://CRAN.R-project.org/package=bootComb).

**Key Features**

- bootComb derives confidence intervals with the required coverage for parameters that are computed from independent parameter estimates for which confidence intervals are reported.
- Includes auxilliary functions for 6 common distributions (beta, normal, exponential, gamma, Poisson and negative binomial) to derive best-fit distributions (and their sampling functions) for parameters given their reported confidence intervals.
- R package: open-source, easy-to-use, platform independent.
- Stable version hosted on CRAN: https://CRAN.R-project.org/package=bootComb
- Latest development version available from GitHub: https://github.com/gitMarcH/bootComb

## Introduction

### Motivation

In epidemiological research, the need to combine several estimated parameters is not unusual. The impact of study or facility-based limitations on parameter estimates is well-known^1^ and common adjustment factors include the probability of seeking healthcare or of receiving a diagnostic test (both in the case of facility-based estimates), the incidence or prevalence of a related condition (in the case of a conditional disease prevalence/incidence), or the operational characteristics of the diagnostic test (in the case of imperfect diagnostic tests). A recent example includes the estimation of typhoid incidence^1^ where a Bayesian model was used to derive adjustment factors. While usually easy to combine point estimates, it is often difficult to obtain a valid confidence interval (CI) for the combined parameter.

The development of bootComb was motivated by two real-world examples:

- Obtaining a 95% CI for hepatitis D virus (HDV) prevalence from the reported estimates and 95% CIs for the conditional prevalence of hepatitis D among hepatitis B surface antigen (HBsAg) positive patients and the prevalence of HBsAg.^2^
- Adjusting the seroprevalence estimate obtained from a novel antibody test for SARS-CoV-2 for the estimated sensitivity and specificity of this test.^3^

In both applications, the parameter of interest was the unconditional (HDV example) or the adjusted (SARS-CoV-2 example) prevalence, not the raw, directly measured estimate and in each case, multiple independently estimated parameters had to be combined via a known mathematical function. However, it was not evident how to derive a corresponding CI.

In public health applications, CIs are as important for policy makers than the central point estimates. CIs are needed for the adjusted incidence or prevalence parameters, not for the raw, unadjusted estimates. Given recent guidelines^4^ for nuanced discussion of the full range of values within estimated CIs rather than just a focus on point estimates and p-values, there is a large need for CIs with correct coverage and this is where bootComb provides a simple-to-use tool to propagate uncertainty from all estimates.

While in both examples above all parameters are probability parameters, the algorithm is general: it can be used for arbitrarily complex functions to combine an arbitrary number of parameters, each with an arbitrary distribution (provided it can be sampled from). 

### Context relative to previously existing software

For some situations, e.g. the sum of two normally distributed, exact solutions exist. There are software implementations for the example of adjusting a prevalence estimate for the sensitivity and specificity of the diagnostic test (e.g. *Reiczigel et al*^5^, or https://larremorelab.github.io/covid-calculator26). The former of these assumes that sensitivity and specificity are known exactly. For specific applications, a Bayesian model^6^ or non-parametric bootstrapping^7^ will propagate uncertainty from all parameters but implementation of such approaches requires substantial statistical programming expertise.

Crucially, all of the above are for specific applications and the author is not aware of a software implementation for the general problem of deriving CIs for arbitrary functions of an arbitrary number of parameter estimates each with an arbitrary probability distribution.

## Implementation

bootComb is a package for the statistical computation environment R^8^ and its source code is written in R. bootComb is available from the Comprehensive R Archive Network (https://CRAN.R-project.org/package=bootComb) and can be installed within R by typing the following at the R console: install.packages(‘bootComb’). Source code and the latest development version are available from GitHub (https://github.com/gitMarcH/bootComb). To compute highest density intervals bootComb makes use of the R package HDInterval^9^. If this is not installed, bootComb falls back on the percentile method.

### The algorithm

Assume that a parameter of interest ϕ is computed from k=2,3,.. parameters $\theta =(\theta_{1},\ldots ,\theta_{k})$ using a function g: $\phi =g(\theta_{1},\ldots ,\theta_{k})$. Assume that for each parameter $\theta_{j}$, j=1,…,k, an estimate ${\hat{\theta }}_{j}$ with an (1-α)⋅100% CI $\left[{\hat{\theta }}_{l,j},{\hat{\theta }}_{u,j}\right]$ is reported.

An estimate for ϕ is obtained by computing $\hat{\phi }=g\left({\hat{\theta }}_{1},\ldots ,{\hat{\theta }}_{k}\right)$, but it is less obvious how to derive a CI for $\hat{\phi }$ with correct coverage (1-α)⋅100%. For example, for independent parameter estimates, the naively computed interval $\left[g\left({\hat{\theta }}_{l,1},\ldots ,{\hat{\theta }}_{l,k}),g({\hat{\theta }}_{u,1},\ldots ,{\hat{\theta }}_{u,k}\right)\right]$ will be too wide.

Writing $\Theta_{j}$ for the estimator for $\theta_{j}$, and assuming $\Theta_{j}∼F_{j}$, where $F_{j}$ is some parametric distribution, for each parameter estimate ${\hat{\theta }}_{j},j=1,\ldots ,k$, we can estimate a probability distribution ${\hat{F}}_{j}$ from the reported CI $\left[{\hat{\theta }}_{l,j},{\hat{\theta }}_{u,j}\right]$ and then use parametric bootstrap sampling to obtain an approximate CI for $\hat{\phi }$ with the required coverage.

The general algorithm is given below:

- For j=1,…,k, estimate a distribution function ${\hat{F}}_{j}$ for the estimate $\Theta_{j}$ from $\left[{\hat{\theta }}_{l,j},{\hat{\theta }}_{u,j}\right]$.
- Assuming that the parameters $\theta_{1},\ldots ,\theta_{k}$ (and their estimates ${\hat{\theta }}_{1},\ldots ,{\hat{\theta }}_{k}$) are independent, obtain B bootstrap samples ${\hat{\theta }}^{\left(b\right)}$, b=1,…,B, for $\hat{\theta }=({\hat{\theta }}_{1},\ldots ,{\hat{\theta }}_{k})$ by sampling ${\hat{\theta }}_{j}^{\left(b\right)}∼{\hat{F}}_{j}$, j=1,…,k independently.
- For each bootstrap sample b, compute ${\hat{\phi }}^{\left(b\right)}=g\left({\hat{\theta }}_{1}^{\left(b\right)},\ldots ,{\hat{\theta }}_{k}^{\left(b\right)}\right)$, b=1,…,B.
- Obtain a (1-α)⋅100% CI $\left[{\hat{\phi }}_{l},{\hat{\phi }}_{u}\right]$, using either the percentile^9^ or the highest density interval^10^ methods on the empirical distribution for $\hat{\phi }$ given by the sample ${\left\{{\hat{\phi }}^{\left(b\right)}\right\}}_{b=1,\ldots ,B}$.

### Method for deriving CIs from a sample

As an alternative to the common percentile method^10^, the highest density interval (HDI)^11^ can be used to derive the required CI. The advantage is that this is the narrowest interval with the desired coverage and that the probability density estimated from the bootstrap sample is always higher or equal inside the interval compared to outside it. One caveat is that the HDI may not be a single interval but a set of intervals if the density is multimodal. In this case the single interval returned by bootComb may be too wide and users need to inspect histograms of the sampled combined parameter to check for multimodality when using bootComb with method=’hdi’. The default is method=’quantile’ which implements the percentile method.

## Use

This section contains worked examples for the two applications from the Introduction section. The main computational routine, bootComb(), is general and not limited to probability parameters as is the case here where the beta distribution, a natural candidate for probability parameters, was used.

### Hepatitis D virus prevalence in the general population

A pre-condition for hepatitis D virus (HDV) infection is hepatitis B virus (HBV) infection. To assess HBV prevalence, study participants can be tested for the presence of surface antigen of the hepatitis B virus (HBsAg). To assess HDV prevalence, one can test for the presence HDV-specific immunoglobulin G antibodies (anti-HDV).

HDV is rare and, since it is conditional on HBV, most studies report the prevalence of anti-HDV among HBsAg-positive patients. Stockdale *et al*^2^ conducted a systematic review to estimate the global conditional prevalence ${\hat{p}}_{\mathrm{aHDV}|\mathrm{HBsAg}}$ and, using estimates of HBsAg prevalence ${\hat{p}}_{\mathrm{HBsAg}}$ reported by the World Health Organization (WHO), to derive ${\hat{p}}_{\mathrm{aHDV}}$: ${\hat{p}}_{\mathrm{aHDV}}={\hat{p}}_{\mathrm{aHDV}|\mathrm{HBsAg}}\cdot {\hat{p}}_{\mathrm{HBsAg}}$. The CI for ${\hat{p}}_{\mathrm{aHDV}}$ for the global population, reported in Table 2 in Stockdale *et al*^2^ was derived using the bootComb algorithm:

- ${\hat{p}}_{\mathrm{HBsAg}}=3.5\%$ with 95% CI (2.7%, 5.0%).
- ${\hat{p}}_{\mathrm{aHDV}|\mathrm{HBsAg}}=4.5\%$ with 95% CI (3.6%,5.7%).

**library**(bootComb)

*# find best-fit beta distribution for the reported CIs*

dist1<-**getBetaFromCI**(qLow=0.027,qUpp=0.050,alpha= 0.05) *# p_HBsAg*

dist2<-**getBetaFromCI**(qLow=0.036,qUpp=0.057,alpha= 0.05) *# p_aHDV|HBsAg*

distList<-**list**(dist1**$**r,dist2**$**r)

*# combination function*

combFun<-**function**(pars){pars[[1]]*pars[[2]]}

*# point estimate of the combined parameter*

p_aHDV<-**combFun**(**c**(0.035,0.045))

**print**(p_aHDV)

## [1] 0.001575

*# 95% CI*

ci<-**bootComb**(distList=distList,combFun=combFun) **$**conf.int

**print**(ci)

## 2.5% 97.5%

## 0.001144280 0.002468875

We obtain the estimate ${\hat{p}}_{\mathrm{aHDV}}=0.16\%$ with 95% CI (0.11%,  0.25%).^1^

The estimated beta distributions for the two prevalences in this example have parameters α=39.62,β=1012.19 and α=69.60,β=1445.16. These prevalences can be interpreted as having been estimated from samples of sizes approximately 40 + 1012 = 1052 and 70 + 1445 = 1515, respectively. This can be used to check, via simulation, the coverage of the CI. Using the bootComb function simScenProductTwoPrevs by running simScenProductTwoPrevs(B = 1000, p1 = 0.035, p2 = 0.045, nExp1 = 1052, nExp2 = 1515, alpha = 0.05) shows that the 95% CI has 95.1% coverage, with a 95% CI of (93.6%,96.4%) from *N* = 1000 simulations.

### Sars-CoV-2 seroprevalence adjusted for test sensitivity and specificity

Chibwana *et al*^3^ report the surprisingly high SARS-CoV-2 seroprevalence and associated low morbidity in health workers in Blantyre, Malawi. Writing π for the seroprevalence of SARS-CoV-2, out of 500 study participants, 84 tested positive for SARS-CoV-2 antibodies: ${\hat{\pi }}_{\mathrm{raw}}=16.8\%$ with exact binomial 95% CI (13.6%,20.4%).

The immunological assay used in the study was novel and had been assessed in a limited number of samples as follows^3^^,^^12^:

- sensitivity: 238 of 270 known positive samples tested positive ${\hat{p}}_{\mathrm{sens}}=88.1\%$, 95% CI (83.7%,91.8%);
- specificity: 82 of 88 known negative samples tested negative ${\hat{p}}_{\mathrm{spec}}=93.2\%$, 95% CI (85.7%,97.5%).

Given that the test has sensitivity and specificity below 100%, and the substantial uncertainty of both estimates, it is important to adjust seroprevalences estimated using this test. This is a common situation, e.g. molecular tests are increasingly developed to replace culture-based assays.

Writing $p_{\mathrm{sens}}=P(T|D)$ and $p_{\mathrm{spec}}=P(\bar{T}|\bar{D})$ where T is the event of testing positive, D is the event of being seropositive, and $\bar{T},\bar{D}$ are the complements of T,D, the measured seroprevalence ${\hat{\pi }}_{\mathrm{raw}}$ is related to the estimate of the actual seroprevalence $\hat{\pi }$:

$${\hat{\pi }}_{\mathrm{raw}}=\hat{\pi }\cdot P(T|D)+(1-\hat{\pi })\cdot P(T|\bar{D})$$

We can derive an equation to adjust the measured seroprevalence for the assay’s sensitivity and specificity:

$$\hat{\pi }=\frac{{\hat{\pi }}_{\mathrm{raw}}-P(T|\bar{D})}{P(T|D)-P(T|\bar{D})}=\frac{{\hat{\pi }}_{\mathrm{raw}}+{\hat{p}}_{\mathrm{spec}}-1}{{\hat{p}}_{\mathrm{sens}}+{\hat{p}}_{\mathrm{spec}}-1}$$

where we have substituted the estimated sensitivity and specificity in the expression on the right-hand side.

To summarize, we have three parameter estimates (${\hat{\pi }}_{\mathrm{raw}},{\hat{p}}_{\mathrm{sens}},{\hat{p}}_{\mathrm{spec}}$), their 95% CIs and a functional form to derive the actual parameter of interest ($\hat{\pi }$). With this we can use bootComb, which includes a dedicated function, adjPrevSensSpecCI, for this problem:

**library**(bootComb)

**adjPrevSensSpecCI**(

   prevCI=**binom.test**(x=84,n=500)**$**conf.int, *# 95% CI observed prevalence*

   sensCI=**binom.test**(x=238,n=270)**$**conf.int, *# 95% CI observed sensitivity*

   specCI=**binom.test**(x=82,n=88)**$**conf.int, *# 95% CI observed specificity*

   method=’hdi’,

   prev=84**/**500,*# observed prevalence*

   sens=238**/**270, *# observed sensitivity*

   spec=82**/**88) *# observed specificity*

## $estimate

## [1] 0.1227324

##

## $conf.int

## lower upper

## 0.03926495 0.19013038

## attr(‘credMass’)

## [1] 0.95.

**Figure 1:**
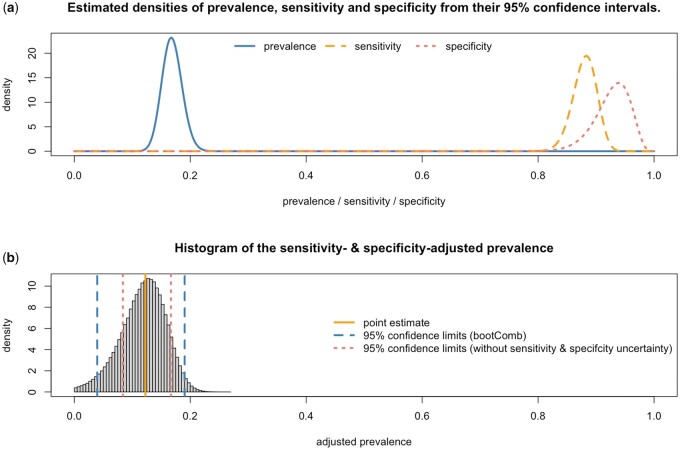
(a) Best-fit beta distributions for the unadjusted seroprevalence, sensitivity and specificity from their 95% CIs. (b) Histogram of the adjusted prevalence values obtained from the bootstrapped values for prevalence, sensitivity and specificity

This yields the estimate $\hat{\pi }=12.3\%$ with 95% CI (3.9%,19.0%). Had the uncertainty in the sensitivity and specificity been ignored, the 95% CI would have been (8.4%,16.7%). Figure 1 illustrates this example. The bootComb package provides a function, simScenPrevSensSpec, for running simulations for this particular application which allows estimation of the actual coverage of the CIs: simScenPrevSensSpec(p = 0.1227, sens = 0.881, spec = 0.932, nExp = 500, nExpSens = 270, nExpSpec = 88, B = 1000). The bootComb 95% CI has estimated 95.3% coverage, with 95% CI (93.8%, 96.5%), whereas ignoring the uncertainty in sensitivity and specificity yields only 75.7% coverage, 95% CI (72.9%, 78.3%) (both from *N* = 1000 simulations; bootComb computes coverage for the latter interval if the argument assumeSensSpecExact=TRUE is passed to the function simScenPrevSensSpec).

## Discussion

This paper presents bootComb, an R package to derive CIs for arbitrary functions of an arbitrary number of estimated parameters, where each parameter estimate follows an arbitrary distribution function. bootComb samples from the empirical distributions of the input parameter estimates and uses either the percentile or high density interval (HDI) method to obtain a CI for the parameter of interest.

The applicability of this R package is wide but has one important limitation: in its current version, bootComb assumes all parameter estimates to be independent. Where this is not the case, the CIs computed by bootComb could have incorrect coverage. In the adjusted seroprevalence example, the three parameters are not independent, even though they were estimated from independent samples. This is apparent in a small number of adjusted prevalences $\hat{\pi }<0$ that were obtained. In most applications, especially for large sample sizes, this error is negligible; in the seroprevalence example this is confirmed by the correct coverage of the CI. In cases where independence is not met, bootComb will err on the side of being too conservative, resulting in overly wide CIs. This is preferable to not correctly propagating uncertainty and reporting CIs with coverage below the targeted level. For many, if not most, situations, the independence assumption will hold (when parameters are obtained from independent studies without direct dependence between the combined parameters) or its violation will only negligibly affect the coverage of the resulting CIs (as in the adjusted seroprevalence example). Nevertheless, future versions of the package will aim to support a limited number of joint distributions. For more complicated dependence situations, custom modelling approaches will be needed.

bootComb provides an easy-to-use tool to the applied epidemiologist faced with the need to combine several independent parameter estimates. At the time of publication, the most recent version of bootComb was 1.0.1. R version 4.0.2 and HDInterval version 0.2.2 were used for computations in this paper.

## Funding

This work was supported by a Wellcome Trust Strategic Award to Malawi—Liverpool—Wellcome Trust Clinical Research Programme [grant: 206545/Z/17/Z].

## Data availability

The data underlying this article are available in the article.
